# Change in p53 nuclear localization in response to extracellular matrix stiffness

**DOI:** 10.1002/SMMD.20240026

**Published:** 2024-11-17

**Authors:** Yan Zu, Jing Du, Yipu Xu, Mengying Niu, Canlin Hong, Chun Yang

**Affiliations:** ^1^ Institute of Biomechanics and Medical Engineering School of Aerospace Engineering Tsinghua University Beijing China; ^2^ Wenzhou Institute University of Chinese Academy of Sciences Wenzhou Zhejiang China; ^3^ Key Laboratory for Biomechanics and Mechanobiology of Ministry of Education Beijing Advanced Innovation Center for Biomedical Engineering School of Biological Science and Medical Engineering Beihang University Beijing China; ^4^ Department of General Dentistry and Emergency Dental Care Beijing Stomatological Hospital Capital Medical University Beijing China

**Keywords:** chondrocytes fate, ECM stiffness, miR‐532, p53 subcellular localization

## Abstract

Chondrocytes are commonly applied in regenerative medicine and tissue engineering. Thus, the discovery of optimal culture conditions to obtain cells with good properties and behavior for transplantation is important. In addition to biochemical cues, physical and biomechanical changes can affect the proliferation and protein expression of chondrocytes. Here we investigated the effect of extracellular matrix stiffness on mouse articular chondrocyte phenotype, growth, and subcellular p53 localization. Chondrocytes were seeded on collagen‐coated substrates varying in elasticity: 0.5 and 100 kPa. Immunocytochemical staining and immunoblotting showed that a softer substrate significantly increased p53 nuclear localization in chondrocytes. Furthermore, we identified microRNA‐532 (miR‐532) as a potential p53 target gene to influence cell function, indicating a new target for tissue engineering. These findings provide insight into the influence of physical cues on cell phenotype maintenance and could help improve understanding of cartilage‐related pathologies such as osteoarthritis.

## Graphical abstract

The expression of p53 in chondrocytes affects a variety of cellular processes, including metabolism, senescence, autophagy, apoptosis, DNA repair, and suppression of the cell cycle. We investigated the influence of extracellular matrix stiffness on mouse articular chondrocyte phenotype, growth, and subcellular p53 localization. The result showed that a softer substrate significantly increased p53 nuclear localization in chondrocytes. Furthermore, we identified microRNA‐532 (miR‐532) as a potential p53 target gene to influence cell function, indicating a new target for tissue engineering. These findings provide insight into the influence of physical cues on cell phenotype maintenance and could help improve the understanding of cartilage‐related pathologies such as osteoarthritis.

**Key points**

- Physical and biomechanical changes affect the proliferation and protein expression of chondrocytes.
- Two substrates with different elasticity were fabricated and the elasticity was 0.5 and 100 kPa, respectively.
- The softer substrate increased p53 nuclear localization in chondrocytes.
- MicroRNA‐532 (miR‐532) can function as a potential p53 target gene to influence cell function for tissue engineering.

## 1 INTRODUCTION

Tissue cells perceive and respond to chemical and physical signals from the extracellular environment.^1^, ^2^, ^3^ Extracellular matrix (ECM) stiffness is proposed as equally important as biochemical cues in this regard and takes part in regulating cellular phenotypes and functions, for example, proliferation, differentiation, migration, and apoptosis.^4^, ^5^, ^6^, ^7^, ^8^, ^9^, ^10^ Previously, a study demonstrated that mesenchymal stem cells are particularly sensitive to changes in ECM stiffness. On compliant matrix (1 kPa), mesenchymal stem cells differentiated into a neural lineage. While on a stiffer matrix (10 and 100 kPa, respectively), they differentiated into myotube and bone cell lineages.^4^

As a tissue with mechanical function, cartilage is commonly used in exploring the physical cues of the extracellular environment.^11^ The physical properties of a cartilage matrix change dramatically at both the spatial and temporal scales to facilitate its mechanical and biological functions.^12^ However, we can utilize different biomaterials and techniques to alter the stiffness of cartilage ECM in vitro for research.^13^ Indeed, the stiffness of the cartilage ECM can vary up to three‐fold during the transition from fetal to adult cartilage^14^ and when moving from the pericellular to the interterritorial matrix.^15^ Moreover, such changes in stiffness have been associated with pathologies. Compared to normal cartilage, the cartilage of patients with late‐stage osteoarthritis (OA) showed a two‐fold increase in matrix stiffness.^16^ It was found that chondrocytes grown on a polyacrylamide (PA) gel of 4 kPa showed decreased proliferation and F‐actin levels but increased type II collagen and aggrecan expression compared to gels of harder stiffness.^17^ Allen et al.^12^ found that chondrogenic marker genes such as *Sox9*, *Col2α1*, and aggrecan were expressed higher in chondrocytes grown on a 500‐kPa PA gel than in those grown on 1100‐ and 200‐kPa gels. Under the physiological conditions, chondrocytes are embedded in the pericellular matrix (PCM), which is a key environmental factor for the cells due to the mechanical property of the matrix. It was observed that the Young's modulus of the enzyme‐isolated PCM (∼1 kPa) was lower than that of the cartilage ECM.^18^ Such a softer matrix is much more similar to the in vivo chondrocyte environment than the stiffness of matrices used in previous studies (in the MPa range). Accordingly, we hypothesized that the stiffness of the cartilage ECM plays a crucial role in the chondrocyte phenotype maintenance and homeostasis, which is possibly by regulating key signaling proteins controlling the cell fate. Thus, chondrocytes were cultured on collagen I‐coated PA gels containing soft and rigid types (0.5 and 100 kPa), respectively, which mimic the stiffness of chondrocytes in vivo environment in young and older individuals, to explore the influence of an elastic stimulus on chondrocyte growth and gene expression.^19^ In particular, we investigated whether ECM stiffness can change p53 activation in chondrocytes, given that p53 and elasticity stimuli share many effectors and take part in many same cellular processes.

P53 is involved in various cellular processes, including apoptosis, proliferation, differentiation, function, and inhibition of angiogenesis.^20^, ^21^ The p53 pathway is influenced by multiple stress states such as heat shock and cancer chemotherapy compounds.^22^, ^23^ However, the effect of elastic stimuli on this pathway has not been well clarified in chondrocytes or other types of cells.

**FIGURE 1:**
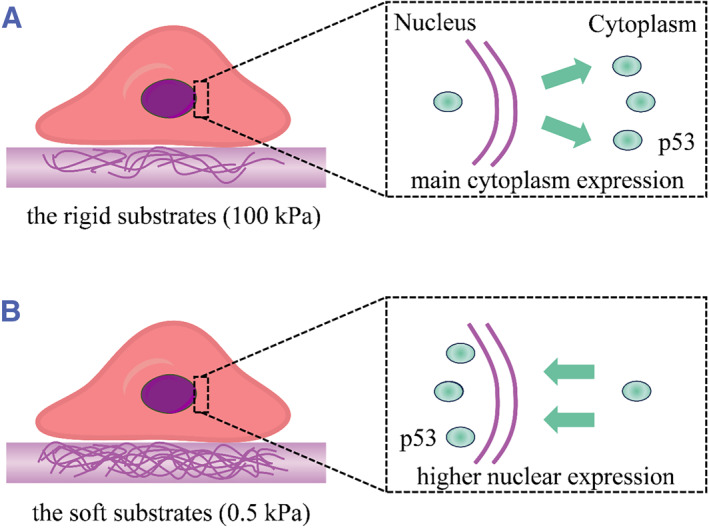
The p53 nuclear localization in chondrocytes grown on 0.5 and 100 kPa substrates. (A) The p53 nuclear localization on the rigid substrates. (B) The p53 nuclear localization on the soft substrates.

We cultured primary murine chondrocytes on 0.5 and 100 kPa substrates and evaluated their proliferation and spreading area, as well as the rate of apoptosis and expression levels of chondrogenesis markers. Further, we determined the p53 nuclear localization in chondrocytes grown on the two substrate types (Figure 1). Through analysis of the downstream genes of p53, we suggest the importance of p53 in regulating the cell fate of chondrocytes in response to elasticity stimuli. Therefore, using the experimental model of chondrocytes, we provide a possible mechanism by which cells convert physical cues to an appropriate biomechanical response.

## 2 METHODS

### 2.1 Cell culture

Chondrocytes were collected from the knees of Kunming mice, which were 1‐day‐old. In short, we isolated articular cartilage prior to extracting chondrocytes using collagenase digestion. These cells were cultured in Dulbecco's Modified Eagle Medium high glucose medium with the addition of 10% fetal bovine serum (FBS), 1% penicillin‐streptomycin, and 1% glutamine. The chondrocytes were put in the incubator at 37°C in 5% CO_2_.

### 2.2 Substrate preparation

Soft and rigid PA gels were fabricated following a published protocol by Engler et al.^4^ In brief, we mixed the bis‐acrylamide and acrylamide with the indicated concentrations, added the mixture to a glass slide to make it polymerized, and covered the gel with Sulfosuccinimidyl‐6‐[4′‐azido‐2′‐nitrophenylamino] hexanoate (Sulfo‐SANPAH; Pierce). After two times of UV exposure for 10 min, PA was washed twice. The collagen I solution (0.2 mg/mL) was added to the sheet and incubated overnight in 4°C freezer. The elasticity moduli of the two kinds of gels were 0.5 kPa (soft) and 100 kPa (stiff), respectively.

### 2.3 Cell proliferation assay

Chondrocytes were collected at passages 2–8, and phosphate‐buffered saline (PBS) was used to remove free proteins. The cell suspension contained 20 million cells/mL in 0.1% FBS/PBS. 1.5 μM carboxyfluorescein succinimidyl ester (CFSE) was added and vortexed gently. The suspension was held for 8 min at room temperature. After the addition of pre‐warmed FBS to the mixture, the chondrocytes were kept in a 37°C water bath for 10 min. Then, PBS was utilized twice to remove the solution. The pretreated cells were then cultured on the rigid or soft substrate for 2 days. Finally, flow cytometry was used to analyze cell proliferation.

### 2.4 Cell apoptosis assay

The chondrocytes were harvested after the cells were seeded on the rigid or soft substrate for 24 h, and the pellets were resuspended in Annexin V conjugate, which was diluted 1:100 in the corresponding binding buffer. In the dark environment, the samples were maintained at room temperature for 20 min. Then they were placed in an ice bucket prior to the addition of propidium iodide (PI) solution with the concentration of 50 μg/mL to each sample. Confocal analysis was conducted following these procedures.

### 2.5 Immunoblotting and antibodies

In brief, we resolved the proteins from the nucleus by sodium dodecyl sulfate polyacrylamide gel electrophoresis. Then, the samples were transferred to the membrane of polyvinylidene fluoride. The blocking buffer was added for 90 min in 5% milk without fat. Primary antibodies of p53 diluted 1:100 (Cell Signaling Technology) or Glyceraldehyde‐3‐Phosphate Dehydrogenase diluted 1:500 from Santa Cruz Biotechnology were used. Anti‐mouse or anti‐rabbit IgG conjugated with horseradish peroxidase was used to incubate the membrane (diluted 1:10,000). Ultimately, an X‐ray processor (Kodak) developed the membranes.

### 2.6 Immunocytochemical staining

The chondrocytes were cultured on two types of slides coated with gels (soft and stiff). After 4 days, 4% paraformaldehyde was used to fix the cells. Then, the blocking buffer was added for 0.5 h. Primary antibodies against p53 (1:50; Cell Signaling Technology), Aggrecan (1:50; Abcam), collagen I (Col I, 1:100), and collagen II (Col II, 1:50; Abcam), were used to incubate the cells. Secondary antibodies such as FITC‐conjugated anti‐mouse IgG, TRITC‐conjugated anti‐goat IgG, or TRITC‐conjugated anti‐rabbit IgG were added after the aspiration of primary antibodies. Chondrocyte nuclei were stained with 4',6‐diamidino‐2‐phenylindole (Sigma) and observed under a confocal microscope (Leica).

### 2.7 Real‐time PCR

The chondrocytes were gathered and Trizol (Invitrogen) was used to isolate the total mRNA. qRT‐polymerase chain reaction (PCR) was conducted to detect microRNA expression using TaqManmiR assay kits (Tiangen) following the manufacturer's protocol. The normalization of levels of miRs using rRNA U6 was taken as a reference.

### 2.8 Luciferase assay

A mixture of reporter plasmid PGL3‐miR‐532 (1 μg) and pRL‐CMV (5 ng) was used to transfect MC‐3T3 cells. After being transfected for 6 h, cells were treated with Nutlin3 or Pifithrin‐α for 18 h. Dual Luciferase Kit (Promega) was utilized to assess the firefly and Renilla luciferase activities. The pupose of Renilla luciferase activity was to normalize the firefly luciferase values in every sample. The data were reported in the form of relative light units.

### 2.9 Statistical analysis

Data were shown as mean ± SEM. Difference between the two experimental groups was calculated using Student's *t*‐test. ANOVA analysis was conducted for multiple comparison. Asterisks were used to indicate the significance: *p < 0.05; **p < 0.01.

## 3 RESULTS

### 3.1 Collective spreading morphology of chondrocytes on rigid and soft substrates

**FIGURE 2:**
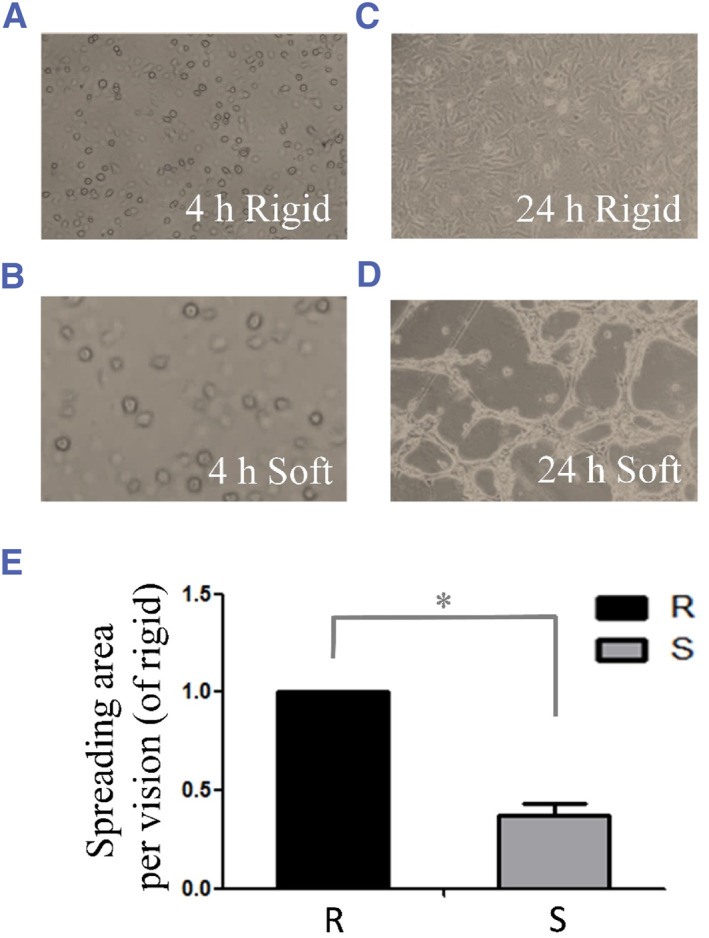
Collective cell spreading morphology of chondrocytes grown on rigid and soft substrates. (A) Chondrocytes were cultured on a 100 kPa rigid substrate for 4 h. (B) Chondrocytes were cultured on a 0.5 kPa soft substrate for 4 h. (C) Chondrocytes were cultured on a 100 kPa rigid substrate for 24 h. (D) Chondrocytes were cultured on a 0.5 kPa soft substrate for 24 h. (E) Quantitative analysis of the spreading area of chondrocytes seeded on the rigid and soft substrates. *p < 0.05.

We seeded cells at an initial density (∼8 × 105 cells per sample) on collagen I‐coated rigid (100 kPa) or soft (0.5 kPa) thick PA gels that were poured on 9.5‐cm^2^ glass dishes. At 4 h after spreading, cells seeded on both the rigid and soft substrates were observed to fill up the free space (Figure 2A,B). We then focused on the collective cell spreading morphology of the cells. As shown in Figure 2C,D, the spreading area per field of view differed according to substrate stiffness after 24 h. When cultured on rigid gels (100 kPa), cells exhibited an expanded and flattened morphology, occupying a larger area of the substrate (Figure 2C). By comparison, chondrocytes grown on soft (0.5 kPa) substrates exhibited a reticular morphology (Figure 2D). At 10× field of microscope view, 10 random images were taken in each sample for quantification, and the experiments were repeated five times. As shown in Figure 2E, the spreading area per field of view was reduced in chondrocytes on the soft substrate after 24 h; the spreading area was 38.4 ± 8.1%, which was significantly smaller than that observed on the rigid substrates.

### 3.2 Proliferation and apoptosis of chondrocytes grown on rigid and soft substrates

**FIGURE 3:**
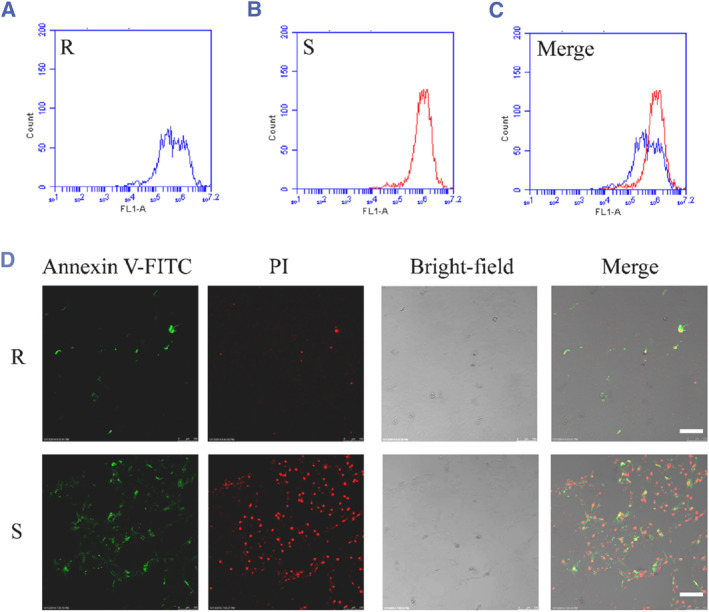
Proliferation and apoptosis of chondrocytes grown on rigid and soft substrates. (A) The fluorescence of chondrocytes seeded on 100 kPa rigid substrate on day 2. (B) The fluorescence of chondrocytes seeded on 0.5 kPa soft substrate on day 2. (C) Merged image of (A and B). (D) Chondrocytes grew on the rigid and soft substrate for 24 h following the determination of Annexin V and PI by analysis of the fluorescence image. Scale bar: 30 μm. PI, propidium iodide.

Considering the difference in the collective spreading morphology of chondrocytes growing on rigid and soft substrates, we next evaluated the effect of substrate type on cell proliferation and apoptosis. To evaluate the influence of elasticity on proliferation, a CFSE assay was performed after the cells reached confluence on day 2. To explore cell proliferation, the flow cytometry result of CFSE‐labeled cells is an approved experimental technique because the CFSE fluorescence in daughter cells halves progressively following every cell division. The original CFSE histograms on day 2 were used to evaluate the number of complete cell divisions for analysis. Figure 3A,B show the number of cells on the rigid (blue line) and soft substrate (red line) at a given fluorescence intensity, respectively. The CFSE fluorescence intensity on the rigid substrate showed two cell division peaks, whereas only one peak was observed for cells cultured on the soft substrate, which indicates chondrocytes on rigid substrate proliferated more times than those on the soft substrate (Figure 3C). The mean fluorescence intensity was significantly decreased on the rigid substrate compared with the soft one, which demonstrated that the soft substrate decreased the proliferation of chondrocytes than the rigid substrate. Moreover, the immunostaining results showed that the soft substrates induced apoptosis of chondrocytes (Figure 3D). Therefore, substrate stiffness has a significant influence on chondrocyte proliferation and apoptosis.

### 3.3 Maintenance of the chondrocyte phenotype on the soft substrate

**FIGURE 4:**
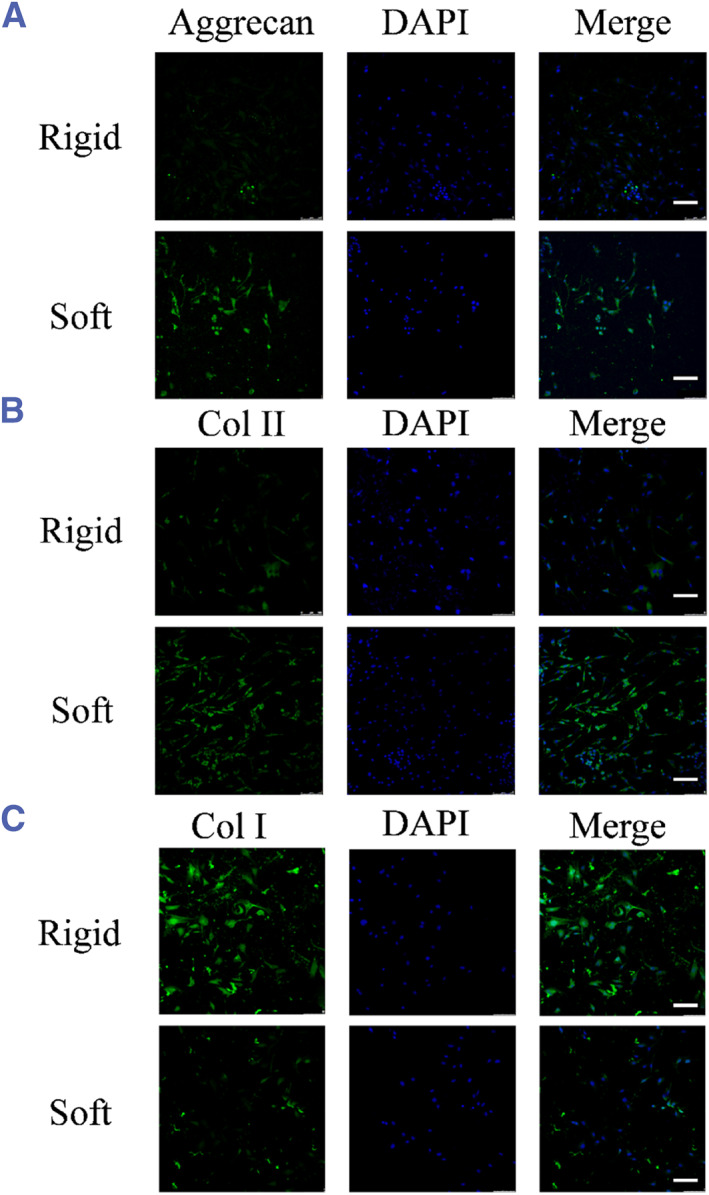
ECM stiffness regulates the maintenance of chondrocyte phenotype. (A) The determination of aggrecan expression in chondrocytes on rigid and soft ECM for 4 days by immunocytochemical staining. (B and C) The determination of Col I and Col II expression in chondrocytes on rigid and soft ECM for 4 days after immunocytochemical staining. Scale bar: 30 μm. ECM, extracellular matrix.

The de‐differentiation of chondrocytes in monolayer culture is the main problem in in vitro cell culture for chondrocytes used in tissue engineering. Thus, our investigation focused on the influence of substrate stiffness on the differentiation ability of chondrocytes. The immunostaining results showed that chondrocytes grown on soft substrates exhibited increased aggrecan expression (Figure 4A). Members of the collagen family also showed significantly different regulation according to substrate stiffness, with a decrease in collagen I expression and an increase in collagen II expression on the soft substrate (Figure 4B,C). Therefore, a soft substrate is more suitable for maintaining the chondrocyte phenotype.

### 3.4 Change in p53 nuclear localization in chondrocytes seeded on the soft substrate

**FIGURE 5:**
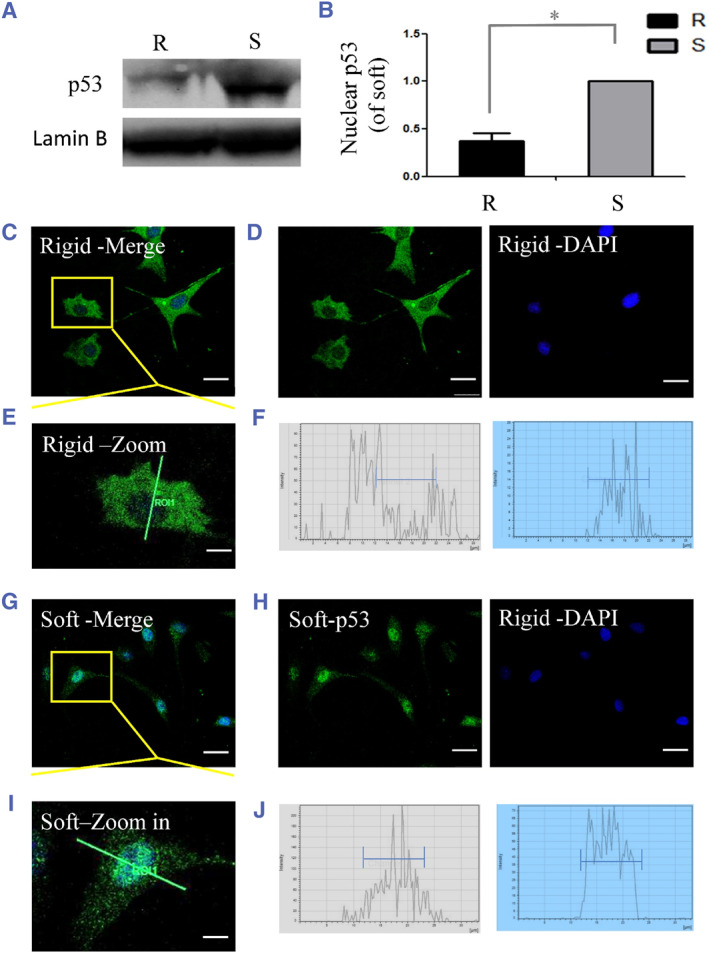
p53 nuclear expression in chondrocytes after 24 h of culture on different Young's moduli matrices (rigid: 100 kPa, soft: 0.5 kPa). (A) Western blot analysis revealed that the substrate stiffness significantly influenced the nuclear expression of p53 in chondrocytes. (B) Quantitative statistical analysis of p53 nuclear expression. *p < 0.05. (C) The merge staining image of cells seeded on the rigid substrate. (D) Cells in (C) stained with p53 (green) and nuclei (blue). (E) The zoomed‐in image of cells seeded on the rigid substrate. The green line across the nuclei of chondrocytes. (F) The fluorescence intensity along the green line in (E). Green shows the intensity of p53 and blue shows the intensity of DAPI. The blue line shows the area of nuclei. (G) The merge staining image of cells seeded on the soft substrate. (H) Cells stained with p53 (green) and nuclei (blue) on the soft substrate. (I) The zoomed‐in image of cells seeded on the soft substrate. The green line across the nuclei of chondrocytes. (J) Fluorescence intensity along the green line in (E). Green shows the intensity of p53 and blue shows the intensity of DAPI on the soft substrate. The blue line shows the area of nuclei. Scale bar: 25 μm. DAPI, 4',6‐diamidino‐2‐phenylindole.

Next, we investigated whether p53 localization was influenced by the changes in substrate stiffness. We isolated the nuclear protein from chondrocytes seeded on soft or rigid substrates after 24 h. The western blotting results showed that chondrocytes grown on the soft substrate had significantly higher p53 nuclear expression (Figure 5A,B). To confirm the western blotting results, p53 nuclear expression was detected by immunofluorescence staining (Figure 5). On the rigid (100 kPa) substrates, the p53 protein was mainly found in the cytoplasm (Figure 5C–F). On the soft (0.5 kPa) substrates, the majority of chondrocytes showed significant p53 nuclear expression (Figure 5G–J). These results confirmed the conception that substrate stiffness influences p53 nuclear expression significantly.

### 3.5 Influence of substrate stiffness on the expression of genes downstream of p53

**FIGURE 6:**
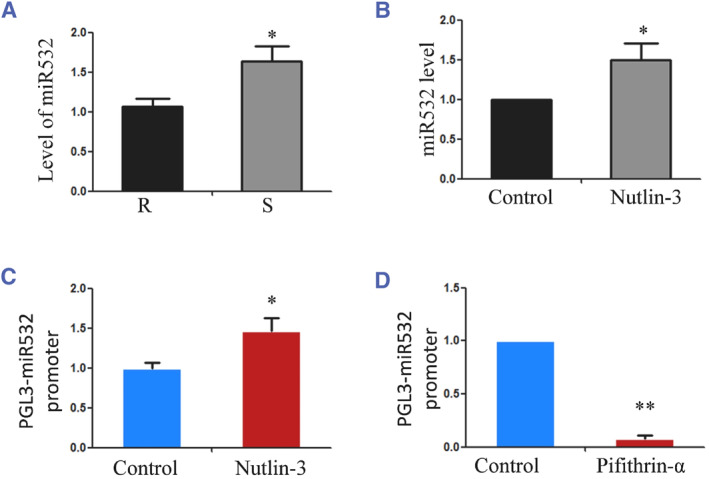
(A) Results from primary chondrocytes seeded on rigid (100 kPa) and soft (0.5 kPa) ECM for 48 h. The miR‐532 expression level was analyzed by real‐time PCR. (B) The miR‐532 level in chondrocytes treated with the p53 activator nutlin‐3 on normal plates. (C) After being transfected with the reporter constructs pGL3‐miR532 promoter, MC‐3T3 cells were then treated with the p53 activator nutlin‐3. (D) After being transfected with the reporter constructs pGL3‐miR532 promoter, MC‐3T3 cells were then treated with the p53 inhibitor pifithrin‐α. *p < 0.05 and **p < 0.01. ECM, extracellular matrix; PCR, polymerase chain reaction.

Given the clear influence of substrate stiffness on p53, we next evaluated its effect on the genes downstream of p53. MiR‐532‐5p is a newly found oncogenic microRNA that influences cell proliferation. A previous study showed that p53 can regulate miR‐532 expression, which may induce an increase in the proliferation of osteoblasts, as shown by the experimental results in Figure 3. Thus, we tested the expression level of miR532 in chondrocytes grown on soft and rigid substrates through real‐time PCR. The results indicated that miR532 expression was upregulated in chondrocytes on a soft substrate (Figure 6A). After the cells were stimulated with the p53 activator nutlin‐3, the expression of miR532 was significantly upregulated (Figure 6B). To further confirm whether p53 is an upstream regulator of miR532, a luciferase reporter containing the miR‐532 promoter with the p53‐binding site was transfected into MC‐3T3 cells. The luciferase assays showed that the p53 inhibitor pifithrin‐α repressed the activity of the miR‐532 promoter reporter (Figure 6C), while the p53 activator nutlin‐3 up‐regulated the activity of the miR‐532 promoter reporter (Figure 6D). Moreover, through analysis of our previous microarray,^6^ we found that the downstream genes of p53 were strongly regulated by substrate stiffness; in particular, *Cx3cl1* and *Btg2* expression was significantly increased on the soft substrate (Table S1). Collectively, these results suggest that p53 transcriptional activity is likely to specifically increase in chondrocytes seeded on soft (0.5 kPa) substrates.

## 4 CONCLUSIONS

P53 is clarified as a 53‐kDa cellular protein and an ultimate tumor suppressor gene.^24^ It is a key regulatory protein responding to amounts of signals and recruiting a series of biochemical activities to trigger multiple biological responses, such as cell cycle arrest and apoptosis.^25^, ^26^ A previous study showed that the expression of p53 enhanced following the apoptotic processes in chondrocytes after being exposed to shear strain,^21^ which serves as an important cue to combine p53 and elasticity stimulation. In our study, the soft (0.5 kPa) extracellular substrates induced the nuclear translocation of transcription factor p53. Through the immunostaining and immunoblotting results, primary murine chondrocytes cultured on 0.5 kPa substrates showed increased p53 nuclear localization compared with those grown on stiffer 100 kPa substrates.

ECM stiffness is demonstrated to influence cell fate in multiple cell types^4^, ^8^, ^12^, ^27^; however, the mechanism and response to elasticity stimuli in chondrocytes have not been systematically studied. We cultured primary murine chondrocytes on soft (0.5 kPa) and rigid (100 kPa) PA gels, which mimic the PCM and ECM of the in vivo environment of chondrocytes. The results showed that chondrocytes cultured on 0.5 kPa substrates have a lower proliferation and spreading area but increased expression of apoptosis and chondrogenesis markers. Interestingly, unlike cellular senescence, increasing the stiffness of the ECM can affect the function of chondrocytes through mechanical transduction, leading to cellular senescence.^28^ However, by analyzing the downstream genes of p53, we suggest the vital role of p53 in manipulating chondrocyte cell fate in response to elasticity stimuli, which in turn has a significant effect on chondrocyte proliferation and apoptosis. The above results indicate that the transcriptional activity of P53 may be specifically increased on soft (0.5 kPa) substrates, which can induce cell apoptosis by activating the expression of pro‐apoptotic genes or inhibiting anti‐apoptotic genes, supporting the finding that apoptosis of chondrocytes is significantly increased in the ECM of the soft (0.5 kPa) substrates. Accordingly, using the experimental model of chondrocytes, we provide a possible mechanism by which cells convert physical cues to a biomechanical influence.

Chondrocytes are the unique resident cells discovered in the cartilage responsible for synthesizing and turnover of ample ECM.^29^ Cultured chondrocytes have been used as a source of cells for transplantation to restore articular tissue.^30^ Thus, keeping chondrocytes in a healthy condition seems a crucial element for maintaining cartilage health and preventing the cartilage degeneration. The regulation of p53, a transcription factor that controls the natural identities of chondrocytes, can be a potential approach for cartilage regeneration. It is possible to obtain chondrocytes with optimal behavior and properties for transplantation by tuning the stimuli molecules and the elastic properties of the substrate, such as combining a high proliferation and chondrogenic phenotype with a soft substrate and a chemical inhibitor of p53 nuclear translocation. A key influence on chondrocyte apoptosis, cellular senescence, and autophagy is the pathological process of OA regulation, which involves the direct induction of p53. Therefore, understanding the characteristics of p53 in chondrocytes is essential for studying the pathogenesis of OA caused by p53 regulation in a series of signaling pathways.^31^, ^32^, ^33^ Furthermore, it might be particularly important to explore the relationship between ECM stiffness and chondrocyte differentiation to achieve a better understanding of OA, which could guide the discovery of novel therapeutic targets.

## AUTHOR CONTRIBUTIONS

Chun Yang conceived the study. Yan Zu and Yipu Xu performed the experiments, analyzed the data, and wrote the manuscript. Mengying Niu, Canlin Hong and Jing Du helped to revise the manuscript.

## CONFLICT OF INTEREST STATEMENT

The authors declare that there are no competing interests.

## ETHICS STATEMENT

This research didn't include any human or animal research.

## Supporting information

Supporting Information S1
