# Supplementary material for: Change in p53 nuclear localization in response to extracellular matrix stiffness
Source: Smart Med. 2024 Nov 17;3(4):e20240026. doi: 10.1002/SMMD.20240026 (PMC11669774; doi:10.1002/SMMD.20240026)
Supplement: Supplementary file 1 — Supporting Information S1 [file SMMD-3-e20240026-s001.docx]

**Change in p53 Nuclear Localization in Response to Extracellular Matrix Stiffness**

**Yan Zu^a,b^**^*^**, Mengying Niu^d^, Canlin Hong^b^, Jing Du^a,c^, Chun Yang^a^**^*^

^a^Institute of Biomechanics and Medical Engineering, School of Aerospace Engineering,

Tsinghua University, Beijing 100084, P.R. China

^b^Wenzhou Institute, University of Chinese Academy of Sciences, Wenzhou, Zhejiang 325000, China

^c^ Key Laboratory for Biomechanics and Mechanobiology of Ministry of Education, Beijing Advanced Innovation Center for Biomedical Engineering, School of Biological Science and Medical Engineering, Beihang University, Beijing, 100083 China

^d^ Department of Anesthesiology, Peking University Third Hospital, Beijing 100191, China

*To whom correspondence may be addressed: E-mail: [zuyan@foxmail.com,](mailto:zuyan@foxmail.com,) yangchun@tsinghua.edu.cn


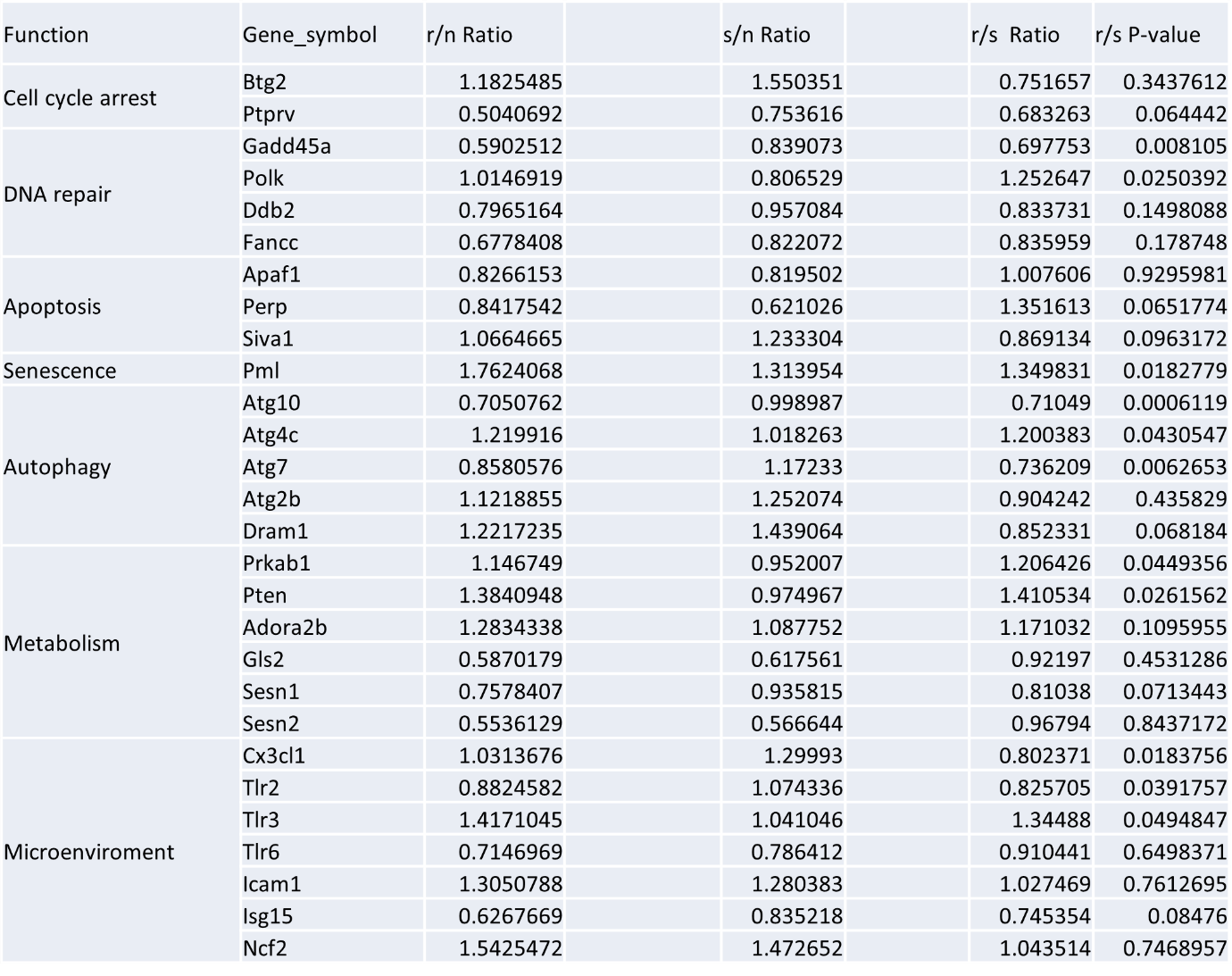


**Table S1.** The expression of downstream genes of p53 regulated by substrate stiffness. (r: rigid substrate, 100 kPa; s: soft substrate, 0.5 kPa)
